# Disinfection by the Fumes of Sulphide of Carbon

**Published:** 1885-02

**Authors:** 


Disinfection by the Fumes of Sulphide of Carbon.—The combustion of this fluid being accompanied by the formation of considerable quantities of sulphurous and carbonic acids, M. P. Vigier has recently recommended its use for the disinfection of rooms and hospitals. Two or three litres of sulphide of carbon were placed in an earthenware jug and ignited. The room is then closed as hermetically as possible, and soon becomes filled by the fumes, which in all probability possess powerful germicide properties. Exact experiments have, however, not yet been made. —*London Medical Record.*

At a recent meeting of the Fairfield County, Conn., Medical Society, Dr. F. M. Wilson, of Bridgeport, demonstrated the anesthetic proprieties of the hydrochlorate of cocaine when administered hypodermically. The *New England Medical Monthly* wishes to claim the honor of priority in this for the profession of Connecticut, New England, the United States, and Dr. Wilson.

